# LRRK2 enhances Nod1/2-mediated inflammatory cytokine production by promoting Rip2 phosphorylation

**DOI:** 10.1007/s13238-016-0326-x

**Published:** 2016-11-09

**Authors:** Ruiqing Yan, Zhihua Liu

**Affiliations:** 1 Key Laboratory of Infection and Immunity of CAS, Institute of Biophysics, Chinese Academy of Sciences, Beijing, 100101 China; 2 University of Chinese Academy of Sciences, Beijing, 100049 China; 3 CAS Center for Excellence in Biomacromolecules, Chinese Academy of Sciences, Beijing, 100101 China

**Keywords:** LRRK2, Nod2, Rip2, NF-κB activation, Inflammation

## Abstract

The innate immune system is critical for clearing infection, and is tightly regulated to avert excessive tissue damage. Nod1/2-Rip2 signaling, which is essential for initiating the innate immune response to bacterial infection and ER stress, is subject to many regulatory mechanisms. In this study, we found that LRRK2, encoded by a gene implicated in Crohn’s disease, leprosy and familial Parkinson’s disease, modulates the strength of Nod1/2-Rip2 signaling by enhancing Rip2 phosphorylation. LRRK2 deficiency markedly reduces cytokine production in macrophages upon Nod2 activation by muramyl dipeptide (MDP), Nod1 activation by D-gamma-Glu-meso-diaminopimelic acid (iE-DAP) or ER stress. Our biochemical study shows that the presence of LRRK2 is necessary for optimal phosphorylation of Rip2 upon Nod2 activation. Therefore, this study reveals that LRRK2 is a new positive regulator of Rip2 and promotes inflammatory cytokine induction through the Nod1/2-Rip2 pathway.

**Electronic supplementary material:**

The online version of this article (doi:10.1007/s13238-016-0326-x) contains supplementary material, which is available to authorized users.

## Introduction

The innate immune system is our first line of defense. It employs pattern recognition receptors (PRRs) to detect a diverse range of pathogen-associated molecular patterns (PAMPs). The NOD-like receptors (NLRs) are one family of such intracellular innate immune receptors. Nod1 and Nod2, two members of the NLR family, are crucial for innate immunity. Upon detecting intracellular bacterial fragments of peptidoglycans, Nod1 and Nod2 initiate proinflammatory responses that depend on gene transcription and other cellular processes, such as autophagy (Philpott et al., 2014; Caruso et al., 2014).

Nod1 and Nod2 share similar domain architecture, comprising carboxyl-terminal leucine-rich repeats (LRRs), a central nucleotide-binding domain, and 1 to 2 amino-terminal caspase recruitment domains (CARDs). Nod1 contains one CARD, whereas Nod2 has two CARDs. The smallest moiety that Nod1 recognizes is D-glutamyl-meso-diaminopimelic acid (iE-DAP), which is present in the cell wall of Gram-negative bacteria and also in selected groups of Gram-positive bacteria. By contrast, Nod2 detects muramyl dipeptide (MDP) that is more ubiquitously present in bacterial cell walls.

Despite detecting different peptidoglycan fragments, Nod1 and Nod2 both initiate a proinflammatory response through a common pathway that depends on activation of NF-κB and MAPK (Philpott et al., 2014; Peterson and Artis 2014). Upon binding peptidoglycan fragments, Nod1 and Nod2 undergo oligomerization, which results in recruitment and activation of Rip2 (Philpott et al., 2014; Caruso et al., 2014; Kobayashi et al., 2002). Rip2-deficient macrophages do not respond to Nod1 or Nod2 ligands (Park et al., 2007; Magalhaes et al., 2011). Activation of Rip2 recruits and activates the TAK1 (TGFβ-activating kinase1)-TAB2-TAB3 complex, which subsequently activates the IKK complex and MAPK pathways (Caruso et al., 2014; Yang et al., 2007). Activated IKK phosphorylates the NF-κB inhibitor IκBα. Phosphorylated IκBα is subsequently modified by polyubiquitination and targeted to the proteasome for degradation (Caruso et al., 2014). The degradation of IκBα allows NF-κB to translocate to the nucleus and influence the expression of downstream target genes. The activation of MAPKs, including ERK, Jun N-terminal kinase (JNK), and P38, also participates in innate responses downstream of Nod1 and Nod2 activation. In addition, ER stress, which occurs during certain bacterial infections, induces inflammation in a Nod1-, Nod2-, and Rip2-dependent manner (Keestra-Gounder et al., 2016). The exact mechanisms by which ER stress activates Nod1 and Nod2 remain to be further determined.

The function of LRRK2 in the immune system is less understood. LRRK2 is better studied in neurons, because multiple mutations of LRRK2 cause familial Parkinson’s disease (PD) (Zimprich et al., 2004; Paisan-Ruiz et al., 2004). It is possible that dysregulation of the immune response contributes to PD development. A role of LRRK2 in immune regulation is suggested by meta-GWAS studies identifying *LRRK2* as a susceptibility locus in Crohn’s disease (CD) and leprosy (Franke et al., 2010; Zhang et al., 2009). In the hematopoietic compartment, the expression of LRRK2 is high in myeloid cells and B cells, but low in T cells (Gardet et al., 2010). The expression of LRRK2 in macrophages is further induced by interferon-γ (Gardet et al., 2010). One study shows that LRRK2 is required for bactericidal activity in macrophages (Gardet et al., 2010). Another study shows that LRRK2 limits the production of inflammatory cytokines in macrophages by negatively regulating NFAT1 in response to zymosan (Liu et al., 2011). LRRK2-deficient mice develop more severe intestinal inflammation in a dextran sodium sulfate-induced colitis model (Liu et al., 2011). However, LRRK2-deficient mice are protected from experimental autoimmune uveitis (EAU) (Wandu et al., 2015). LRRK2 also affects B cell homeostasis and regulates B cell responses upon antigen challenge (Kubo et al., 2016). Thus, the exact role of LRRK2 in immune regulation may depend on specific cell types and the nature of the stimuli.

One study has found that LRRK2 is highly expressed in Paneth cells, and that LRRK2 acts downstream of Nod2 in directing lysozyme sorting in Paneth cells (Zhang et al., 2015). Here we investigated whether LRRK2 also regulates the inflammatory response initiated by Nod2 in macrophages.

## Results

### LRRK2 modulates cytokine production in response to MDP stimulation

**Figure 1:**
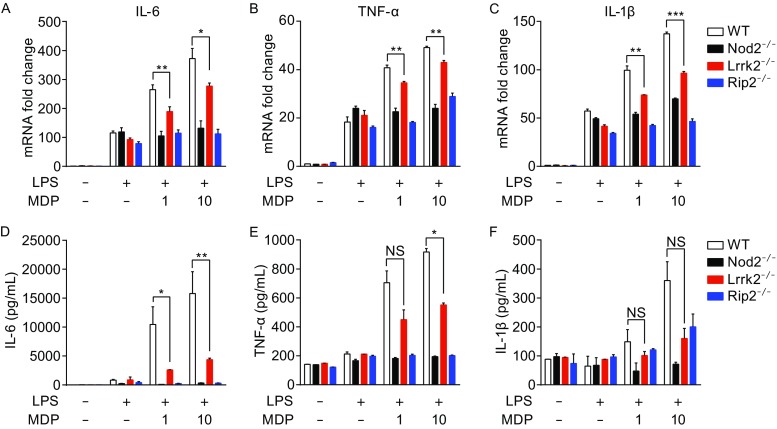
**LRRK2 modulates cytokine production in BMDMs**. (A–C) The relative fold changes of IL-6 (A), TNF-α (B), and IL-1β (C) mRNA transcripts in WT, *Nod2* ^−/−^, *Rip2* ^−/−^ and *Lrrk2* ^−/−^ BMDMs treated with 5 ng/mL LPS and 1 or 10 μg/mL MDP for 4 h, or mock-treated. (D–F) The amounts of IL-6 (D), TNF-α (E), and IL-1β (F) in supernatants from WT, *Nod2* ^−/−^, *Rip2* ^−/−^ and *Lrrk2* ^−/−^ BMDMs treated with 5 ng/mL LPS and 1 or 10 μg/mL MDP for 12 h, or mock-treated. Data are expressed as mean ± s.e.m. *P < 0.05, **P < 0.01, ***P < 0.001, NS denotes not significant, by Student’s *t*-test. Data are representative of three independent experiments (A–F)

Activation of the Nod2 pathway results in the production of inflammatory cytokines. In *in vitro* cultured macrophages, treatment with muramyl dipeptide (MDP) in the presence of a low dose of bacterial lipopolysaccharide (LPS) results in significant production of proinflammatory cytokines, including IL-6, TNF-α and IL-1β (Nakamura et al., 2014). We subjected bone marrow-derived macrophages (BMDMs) from wild-type (WT), *Nod2* ^−/−^, *Lrrk2* ^−/−^ and *Rip2* ^−/−^ mice to such treatments and measured cytokine production. MDP treatment induced dose-dependent increases in the abundance of mRNA transcripts of IL-6, TNF-α and IL-1β in WT BMDMs (Fig. 1A–C). As expected, such increases were diminished in *Nod2* ^−/−^ and *Rip2* ^−/−^ BMDMs (Fig. 1A–C). Interestingly, the level of IL-6, TNF-α and IL-1β mRNA transcripts produced by *Lrrk2* ^−/−^ BMDMs was intermediate amounts between WT and *Nod2* ^−/−^ or *Rip2* ^−/−^ BMDMs (Fig. 1A–C). The effect on cytokine production was further confirmed at the protein level through ELISAs. *Lrrk2* ^−/−^ BMDMs produced significantly less IL-6 and TNF-α compared to WT BMDMs (Fig. 1D and 1E), and a similar trend (though not statistically significant) was observed for IL-1β (Fig. 1F). At the same time, *Lrrk2* ^−/−^ BMDMs produced higher levels of cytokines than *Nod2* ^−/−^ or *Rip2* ^−/−^ BMDMs (Fig. 1D–F). Therefore, we conclude that LRRK2 plays an important role in modulating the production of cytokines in response to MDP, but its role is not as essential as Nod2 or Rip2.

### LRRK2 affects activation of NF-κB and MAPKs in response to MDP

**Figure 2:**
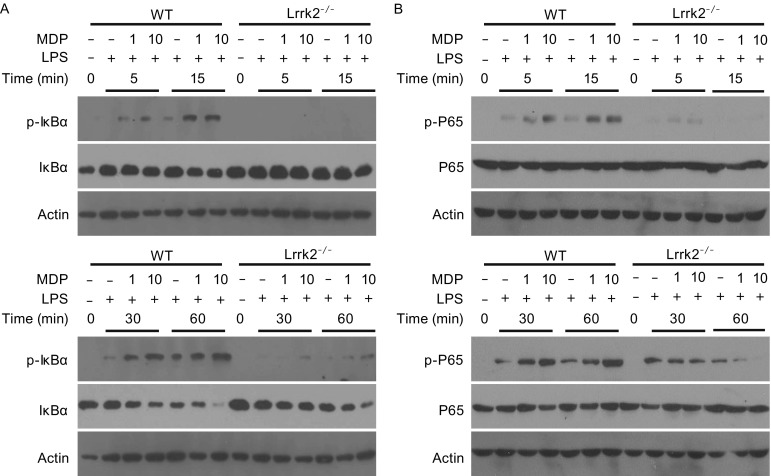
**LRRK2 modulates NF-κB activation in BMDMs**. The levels of phosphorylated IκBα (A) and phosphorylated P65 (B) were monitored in WT and *Lrrk2* ^−/−^ BMDMs treated with 5 ng/mL LPS and 1 or 10 μg/mL MDP for the indicated times. The total amounts of IκBα (A) and P65 (B) were also analyzed. Actin was used as a loading control. Data are representative of three independent experiments (A, B)

To determine how LRRK2 may affect the production of inflammatory cytokines in macrophages in response to MDP, we analyzed the activation of the NF-κB and MAPK pathways, which are known to be responsible for cytokine induction downstream of Nod2-Rip2. The activation of NF-κB can be assessed by monitoring the phosphorylation of the NF-κB inhibitor IκBα by IKK and the degradation of IκBα. Phosphorylation of IκBα was markedly reduced in *Lrrk2* ^−/−^ BMDMs during the treatments, compared to WT BMDMs (Fig. 2A). Consistent with lower levels of phosphorylated IκBα in *Lrrk2* ^−/−^ BMDMs, the disappearance of total IκBα was delayed in *Lrrk2* ^−/−^ BMDMs (Fig. 2A). Furthermore, the phosphorylation of a NF-κB subunit, P65, was markedly reduced in in *Lrrk2* ^−/−^ BMDMs (Fig. 2B). Therefore, LRRK2 deficiency leads to less activation of NF-κB in response to Nod2 engagement in macrophages.

**Figure 3:**
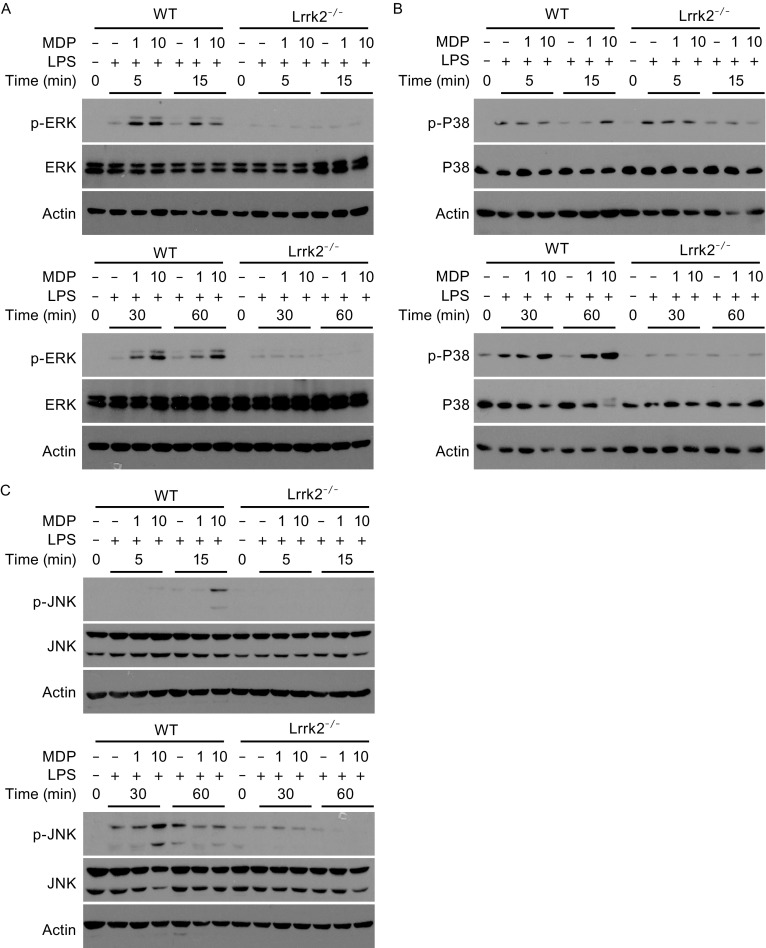
**LRRK2 modulates MAPK pathways in BMDMs**. The levels of phosphorylated ERK1/2 (A), phosphorylated P38 (B) and phosphorylated JNK (C) were determined by immunoblotting of lysates from WT and *Lrrk2* ^−/−^ BMDMs treated with 5 ng/mL LPS and 1 or 10 μg/mL MDP for the indicated times. The total amounts of ERK1/2 (A), P38 (B) and JNK (C) were also analyzed. Actin was used as a loading control. Data are representative of three independent experiments (A–C)

Besides NF-κB, activation of the three MAPK pathways, ERK, P38 and JNK, is also required for the full induction of inflammatory cytokines in response to Nod2 engagement (Philpott et al., 2014; Caruso et al., 2014). We subjected WT and *Lrrk2* ^−/−^ BMDMs to the treatments we used for cytokine induction and analyzed the phosphorylation of the three MAPKs during these treatments. We found that phosphorylation of ERK, P38 and JNK was greatly reduced in *Lrrk2* ^−/−^ BMDMs, compared to that in WT BMDMs (Fig. 3). Therefore, LRRK2 deficiency also reduces the activation of MAPKs in response to Nod2 engagement in macrophages.

### LRRK2 modulates the activation of TAK1

**Figure 4:**
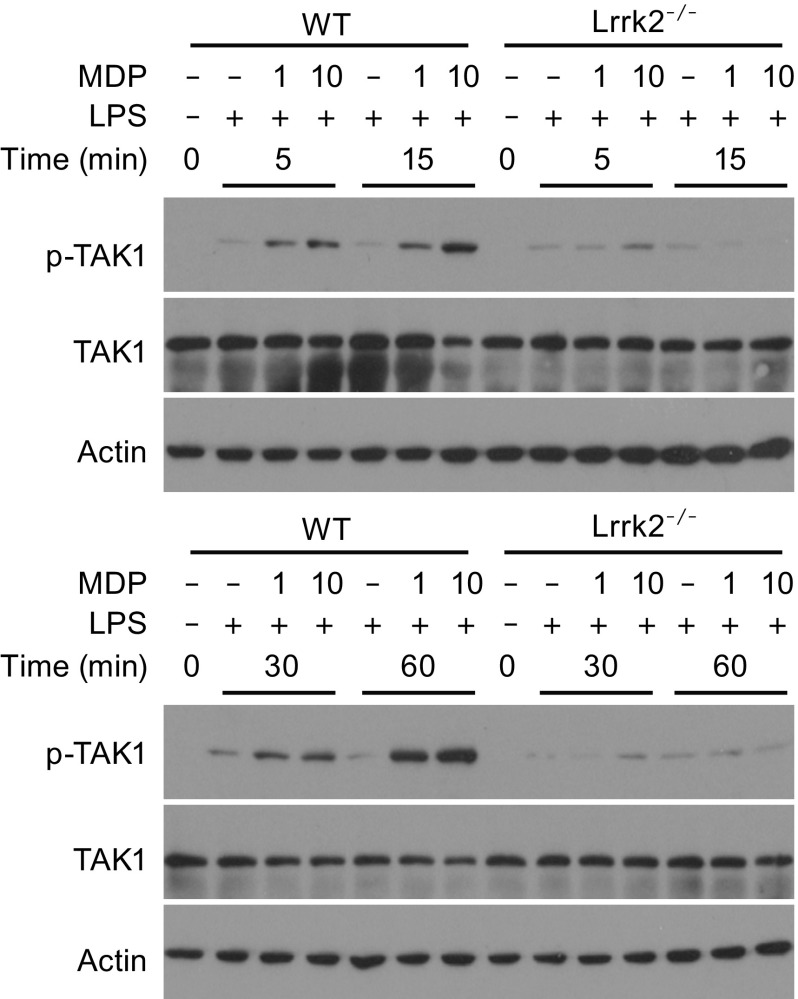
**LRRK2 modulates TAK1 activation in BMDMs**. The amounts of phosphorylated TAK1 and total TAK1 were determined by immunoblotting of lysates from WT and *Lrrk2* ^−/−^ BMDMs treated with 5 ng/mL LPS and 1 or 10 μg/mL MDP for the indicated times. Actin was used as a loading control. Data are representative of three independent experiments

Having demonstrated that LRRK2 affects both NF-κB and MAPK activation, we reasoned that LRRK2 might modulate TAK1 activation, because previous studies have shown that activated TAK1 directly phosphorylates IKK and MAPKs (Philpott et al., 2014; Caruso et al., 2014). We analyzed phosphorylation of TAK1 in WT and *Lrrk2* ^−/−^ BMDMs during the time-course of the combined treatment with LPS and MDP. The phosphorylation of TAK1 was greatly reduced in *Lrrk2* ^−/−^ BMDMs, compared to WT BMDMs (Fig. 4). Therefore, we conclude that LRRK2 promotes TAK1 activation during the activation of the Nod2 pathway.

### LRRK2 enhances activation of Rip2 by promoting its phosphorylation

**Figure 5:**
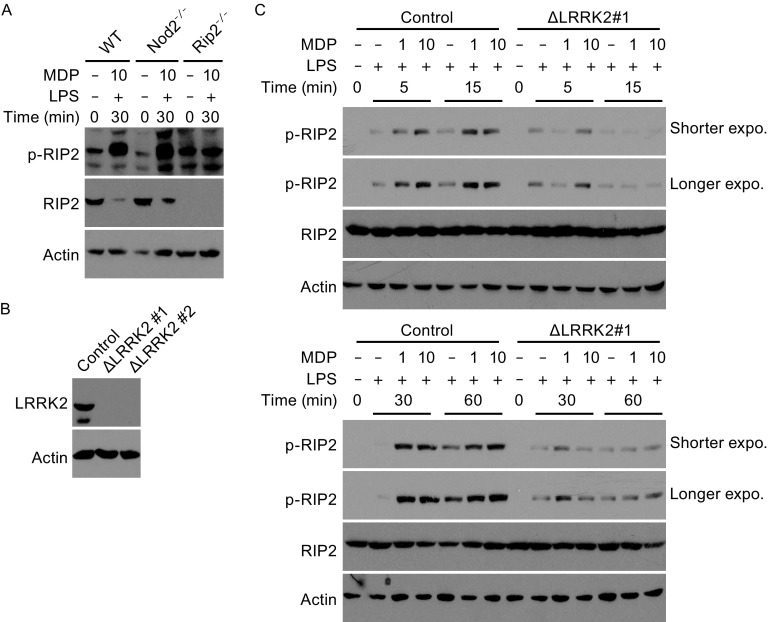
**LRRK2 affects phosphorylation of Rip2 in human macrophages**. (A) Immunoblotting of phosphorylated murine Rip2 (S176) in WT, *Nod2* ^−/−^, and *Rip2* ^−/−^ BMDMs using the commercial antibody against phosphorylated human (S176). BMDMs were treated with 5 ng/mL LPS and 10 μg/mL MDP for 30 minutes or mock-treated. Total Rip2 was immunoblotted as a control. Actin was used as a loading control. (B) Immunoblotting analysis of LRRK2 in control THP-1 cells or THP-1 cells with CRISPR/Cas9 knockout of LRRK2. (C) The amounts of phosphorylated Rip2 and total Rip2 were determined by immunoblotting of lysates from control THP-1 cells and THP-1 cells with LRRK2 knockout (KO #1) treated with 5 ng/mL LPS and 1 or 10 μg/mL MDP for the indicated times. Actin was used as a loading control. Data are representative of three independent experiments (A–C)

We next determined whether LRRK2 affects Rip2 activation downstream of Nod2 engagement. We wanted to analyze the phosphorylation of Rip2 in WT and *Lrrk2* ^−/−^ BMDMs. Upon Nod2 engagement, Rip2 is phosphorylated at Ser176, presumably through the autophosphorylation activity of Rip2 oligomers (Dorsch et al., 2006). The phosphorylation of Rip2 at Ser176 is essential for downstream TAK1 activation (Dorsch et al., 2006). A commercially available anti-phosphorylated Rip2 antibody has been developed and widely used to detect phosphorylated-Ser176 Rip2 of human origin (Dorsch et al., 2006). Although Ser176 is conserved in murine Rip2, the antibody could not be used to recognize endogenous phosphorylated Rip2 in murine cells, since a 51-kd band was also detected in *Rip2* ^−/−^ BMDMs (Fig. 5A). In order to analyze whether LRRK2 affects Rip2 phosphorylation, we generated LRRK2 knockout THP-1 macrophage cells using CRISPR/Cas9. Two independent knockout (KO) lines were generated, ΔLRRK2 #1 and ΔLRRK2 #2, in both of which the expression of LRRK2 was no longer detectable (Fig. 5B). We subjected control and ΔLRRK2 THP1 cells to the MDP/LPS treatment regimen we used on BMDMs. Significant phosphorylation of Rip2 on Ser176 was observed in treated control THP-1 cells (Fig. 5C and 5D); however, in ΔLRRK2 THP1 cells, the phosphorylation of Rip2 was greatly decreased (only the results for ΔLRRK2 #1 cells are presented; similar results were obtained for ΔLRRK2 #2 cells).

**Figure 6:**
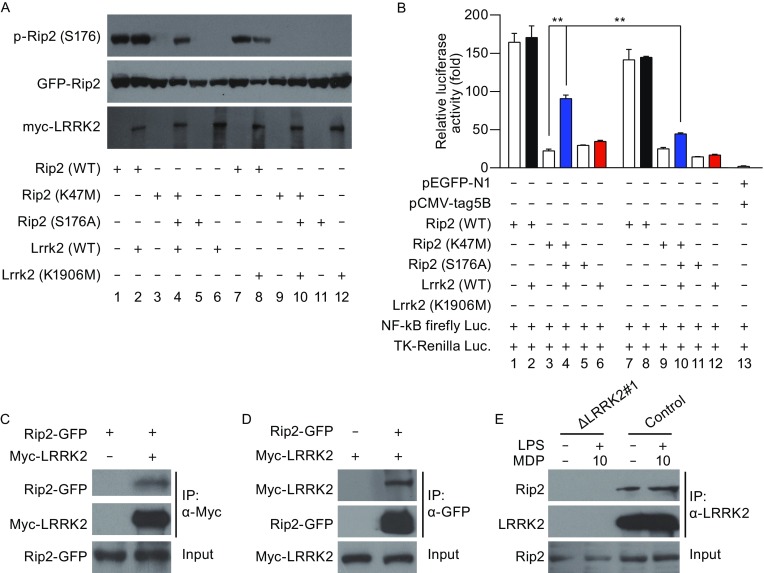
**LRRK2 enhances the phosphorylation of Rip2 at Ser176**. (A) The level of phosphorylated-Ser176 Rip2 was determined by immunoblotting of lysates from HEK293T cells transfected with WT LRRK2 or kinase-dead LRRK2 (K1906M) together with the Rip2 variants WT, K47M or S176A, or empty vectors. The levels of total Rip2 and LRRK2 were analyzed at the same time. (B) Relative NF-κB activity was analyzed using a dual luciferase assay in HEK293T cells transfected with WT LRRK2 or the kinase-dead LRRK2 mutant (K1906M) together with the Rip2 variants WT, K47M or S176A, or empty vectors. Data are expressed as mean ± s.e.m. *P < 0.05, **P < 0.01, ***P < 0.001, NS denotes not significant, by Student’s *t*-test. (C–E) Co-immunoprecipitation analysis of the interaction between overexpressed LRRK2 and Rip2. Tagged LRRK2 and Rip2 were overexpressed in HEK293T cells, immunoprecipitated (IP) with anti-Myc (C) or anti-GFP (D) beads and immunoblotted with designated antibodies. (E) Co-immunoprecipitation analysis of the interaction between endogenously expressed LRRK2 and Rip2. Control THP-1 cells and THP-1 cells with LRRK2 knockout (Δ*LRRK2* #1) were treated with 5 ng/mL LPS and 10 μg/mL MDP for 4 h, or mock-treated, immunoprecipitated with anti-LRRK2 antibody. Rip2 was immunoblotted in input fraction and immunoprecipitated fraction. Data are representative of three independent experiments (A–E)

To study how LRRK2 affects Rip2 phosphorylation, we transfected HEK293T cells with plasmids encoding Rip2 WT, a kinase-dead Rip2 mutant (K47M), or a phosphorylation-null Rip2 mutant (S176A), together with WT LRRK2 or a kinase-dead mutant of LRRK2 (K1906M). The presence of LRRK2 did not alter the phosphorylation of WT Rip2 on Ser176 (Fig. 6A, lane1 and 2). However, LRRK2 greatly enhanced the level of the phosphorylated kinase-dead mutant of Rip2 (K47M) (Fig. 6A, lane 3 and 4). The enhanced phosphorylation of Rip2 K47M depended on the kinase activity of LRRK2, since the kinase-dead version of LRRK2 (K1906M) did not lead to phosphorylation of Rip2 (Fig. 6A, lane 9 and 10). The phosphorylation was indeed at Ser176, since no phosphorylation bands were detected in cells transfected with Rip2 S176A (Fig. 6A, lane 5, 6, 11, 12). To further determine whether the modulation of Rip2 phosphorylation by LRRK2 has a functional outcome, we performed luciferase assays to monitor Rip2-driven NF-κB activation (Fig. 6B). While LRRK2 did not alter the activity of WT Rip2 on NF-κB transcription activity (Fig. 6B, lane 1, 2), LRRK2 drastically increased luciferase activity in cells transfected with kinase-dead Rip2 K47M (Fig. 6B, lane 3, 4). The increase depended on the kinase activity of LRRK2, since the kinase-dead K1906M mutant of LRRK2 did not significantly alter luciferase activity in cells transfected with Rip2 K47M (Fig. 6B, lane 9, 10). We further examined three PD-associated LRRK2 mutants (G2019S, Y1699C, and R1441C) on their abilities in promoting phosphorylation of Rip2 (Fig. S1). We found that G2019S mutant enhanced the phosphorylation of Rip2, however, the mutant of Y1699C or R1441C did not significantly alter the phosphorylation level of Rip2. In comparison, the kinase-dead version of LRRK2, D1994N, completely diminished phosphorylation of Rip2. Therefore, similarly to previous literatures, PD-associated LRRK2 mutants display differential effects in their abilities in Rip2 phosphorylation. Taken together, our results show that LRRK2 enhances Rip2 activity by promoting the phosphorylation of Rip2 at Ser176.

To determine whether LRRK2 physically interacts with Rip2, we performed immunoprecipitation. First, we overexpressed LRRK2 and Rip2 in transfected HEK293T cells, and we found an association between LRRK2 and Rip2 (Fig. 6C and 6D). Second, we examined whether LRRK2 and Rip2 interacted endogenously and whether MDP treatment affected such interaction. We found that anti-LRRK2 pulled down endogenous Rip2 (Fig. 6E), indicating an association of endogenous LRRK2 and Rip2. The association of LRRK2 and Rip2 remained unchanged upon MDP treatment (Fig. 6E). Altogether, our results indicate a scenario that LRRK2 physically interacts with Rip2 and promotes phosphorylation of Rip2.

### LRRK2 modulates cytokine production in response to iE-DAP stimulation

Upon binding their distinct ligands, Nod1 and Nod2 share downstream signaling through Rip2-TAK1 to activate NF-κB transcription activity (Caruso et al., 2014). To determine whether LRRK2 similarly affects Nod1 signaling, we treated *Nod1* ^−/−^, *Lrrk2* ^−/−^, *Rip2* ^−/−^ BMDMs with Nod1 cognate ligand, ie-DAP. As expected, ie-DAP treatment significantly induced the level of IL-6, TNF-α and IL-1β mRNA transcripts, and such induction was largely abolished in *Nod1* ^−/−^ and *Rip2* ^−/−^ BMDMs (Fig. S2A–C). In a comparison, the levels of IL-6, TNF-α and IL-1β mRNA transcripts produced by *Lrrk2* ^−/−^ BMDMs were intermediate between WT and *Nod1* ^−/−^ or *Rip1* ^−/−^ BMDMs (Fig. S2A–C). Therefore, we conclude that LRRK2 similarly promotes cytokine production downstream of Nod1 activation.

### LRRK2 promotes inflammatory cytokine production in ER stress

**Figure 7:**
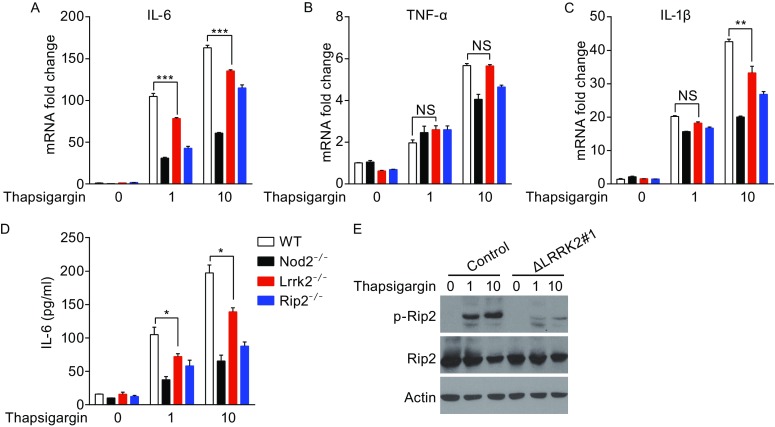
**LRRK2 modulates cytokine production during ER stress**. (A–C) The relative fold changes of IL-6 (A), TNF-α (B), IL-1β (C) mRNA transcript levels in WT, *Nod2* ^−/−^, *Rip2* ^−/−^ and *Lrrk2* ^−/−^ BMDMs treated with 1 or 10 μmol/L Thapsigargin for 24 h, or mock-treated. (D) The amounts of IL-6 in supernatants from WT, *Nod2* ^−/−^, *Rip2* ^−/−^ and *Lrrk2* ^−/−^ BMDMs treated as in (A). Data are expressed as mean ± s.e.m. *P < 0.05, **P < 0.01, ***P < 0.001, NS denotes not significant, by Student’s *t*-test. (E) The levels of phosphorylated Rip2 and total Rip2 were immunoblotted in control THP-1 cells and THP-1 cells with LRRK2 knockout (Δ*LRRK2* #1) treated with 1 or 10 μmol/L Thapsigargin for 24 h. Actin was used as loading controls. Data are representative of three independent experiments (A-E)

ER stress induces proinflammatory cytokine production, which requires the activation of Nod1, Nod2 and Rip2 (Keestra-Gounder et al., 2016). To examine whether LRRK2 also enhances cytokine production in response to ER stress, we treated wild-type (WT), *Nod2* ^−/−^, *Lrrk2* ^−/−^, and *Rip2* ^−/−^ BMDMs with the ER stress inducer thapsigargin, a specific inhibitor of the SERCA channel (Lytton et al., 1991). Thapsigargin treatment significantly induced mRNA transcripts of IL-6, TNF-α, and IL-1β in WT BMDMs, and the induction was reduced in *Nod2* ^−/−^ and *Rip2* ^−/−^ BMDMs (Fig. 7A–C), consistent with the previous study (Keestra-Gounder et al., 2016). Notably, compared with WT BMDMs, IL-6 mRNA transcripts were significantly reduced in thapsigargin-treated *Lrrk2* ^−/−^ BMDMs (Fig. 7A), and this was confirmed by measurement of IL-6 in the supernatant (Fig. 7D). The changes in TNF-α mRNA transcript levels did not reach statistical significance (Fig. 7B). IL-1β mRNA transcript levels were significantly reduced in *Lrrk2* ^−/−^ BMDMs only when thapsigargin was used at a higher dose (Fig. 7C). Thus, LRRK2 enhances cytokine production during ER stress, but to a lesser degree than during MDP treatment. We suspect that the primary reason for this difference is that ER stress induces much less cytokine production than MDP in the first place.

To determine the mechanism how LRRK2 modulates the inflammatory cytokine production in response to ER stress, we analyzed the level of phosphorylated-Rip2 in control and LRRK2-deficient cells. We found that LRRK2 deficiency decreased the level of phosphorylated Rip2 upon thapsigargin treatment (Fig. 7D). Therefore, LRRK2 modulates cytokine production by regulating Rip2 activation during ER stress.

## Discussion

In this study, we uncovered a role of LRRK2 in modulating the innate immune response in macrophages. We found that in *Lrrk2* ^−/−^ BMDMs, cytokine production was attenuated in response to Nod2 or Nod1 ligands. Our biochemical analyses revealed that LRRK2 was required for optimal activation of Rip2, TAK1, NF-κB and MAPKs. Our mechanistic study showed that overexpression of LRRK2 enhanced the phosphorylation of the kinase-dead mutant of Rip2 and boosted the activity of kinase-dead Rip2 in the NF-κB luciferase assay. Finally, we found that LRRK2 also augmented the induction of inflammatory cytokines during ER stress. Thus, our study uncovers a role for LRRK2 in innate immune responses by modulating the strength of Nod2-Rip2-TAK1 signaling.

Because it is indispensable in mediating Nod2 signaling, Rip2 activation has been extensively studied. Ligand engagement by Nod2 leads to Nod2 oligomerization and recruitment of Rip2, and the close proximity of Rip2 leads to autophosphorylation of Ser176. The activation of Rip2 is marked by the covalent attachment of Lys63-linked polyubiquitin to the Lys209 residue (Yang et al., 2007). Several E3 ubiquitin ligases have been suggested to be involved in the activation of Nod2 signaling by promoting Rip2 ubiquitination, including XIAP (X-linked inhibitor of apoptosis protein), TRAF2 (TNFR-associated factor 2), TRAF5, CIAP1 (cellular inhibitor of apoptosis protein 1), CIAP2, and TRAF6 (Philpott et al., 2014; Caruso et al., 2014; Bertrand et al., 2009). Linear ubiquitin chains, which are conjugated through the amino-terminal methionine, also promote activation of the Nod2 signaling pathway by targeting Rip2. XIAP recruits the linear-chain ubiquitin assembly complex (LUBAC) to ligate linear ubiquitin chains to Rip2, and this recruitment and the ubiquitin ligase function of LUBAC are essential for Nod2-dependent responses (Damgaard et al., 2012). Furthermore, polyubiquitylated Rip2 is a substrate for the deubiquitylating enzyme A20, which negatively regulates Nod2 signaling (Hitotsumatsu et al., 2008). Our study identifies LRRK2 as a new regulator of Rip2. Whether LRRK2 directly phosphorylates Rip2 warrants future investigation.

Genetic studies have indicated the involvement of Nod2, Rip2 and LRRK2 in CD and leprosy infection (Zhang et al., 2009; Rioux et al., 2007; Jostins et al., 2012). Our finding that LRRK2 modulates the strength of Nod2 signaling is very interesting. Dysregulated immune responses to commensal bacteria have been generally believed to underlie the development of chronic intestinal inflammation, such as CD. Intestinal flora generate abundant PGN fragments, including Nod2 ligands, which can be sensed by Nod2 either in the intestinal epithelium or in immune cells in the lamina propria. Polymorphisms in Nod2, LRRK2 and Rip2 may all contribute to dysregulated Nod2 signaling. Leprosy is a disease condition caused by chronic infection with *Mycobacterium leprae* and *Mycobacterium lepromatosis* (Suzuki et al., 2012). Nod2 signaling plays a critical role in controlling intracellular infection with bacteria, including mycobacteria. Our finding that LRRK2 modulates Nod2 signaling in macrophages may explain how LRRK2 is involved in leprosy susceptibility.

Besides CD and leprosy, LRRK2 is also involved in Parkinson’s disease. A number of putative substrates for LRRK2 kinase activity have been identified including moesin (Kobayashi et al., 2002; Jaleel et al., 2007) E-BP12 (Keestra-Gounder et al., 2016), β-tubulin (Gillardon, 2009), MAPKK proteins (Hsu et al., 2010; Zhu et al., 2013), ArfGAP13 (Caruso et al., 2014) and Rab10 (Ito et al., 2016). Altered kinase activity has been implicated in a range of cellular processes processes in neurons, including neurite outgrowth, protein translation, mitophagy and *et al* (Jaleel et al., 2007; Imai et al., 2008; Gillardon, 2009; Hsu et al., 2010; Zhu et al., 2013; Stafa et al., 2012; Ito et al., 2016), which may underlie the pathogenesis of LRRK2 mutations in PD. In addition, there are extensive reports in the literature suggesting that dysregulated inflammation is involved in the development of Parkinson’s disease (Leszek et al., 2016). Some authors have even postulated that neural inflammation may underlie neural degeneration (Rocha et al., 2015). Reactive microglia, the resident macrophages in the brain, are enhanced in the hippocampus of PD patients (McGeer et al., 1988). It is rather unclear how inflammation is triggered in the brain, which remains sterile under normal conditions. A recent study has found that ER stress can induce inflammation through Nod proteins (Keestra-Gounder et al., 2016), which could indicate a role of Nod2 signaling in neuroinflammation in neurodegenerative diseases. Our finding that LRRK2 modulates cytokine production during ER stress may offer a new explanation of the involvement of LRRK2 in PD. Together, our results uncover a role of LRRK2 in modulating innate immunity by enhancing the signaling strength of the Nod2-Rip2 pathway. Our study may offer new insights into the role of LRRK2 in multiple diseases.

## Materials and methods

### Mice

*Nod2* ^−/−^, *Nod1* ^−/−^, *Rip2* ^−/−^ and *Lrrk2* ^−/−^ mice on a C57BL/6J background were described previously. All specific-pathogen-free (SPF) mice including WT C57BL/J6 were bred and housed in an AAALAC-accredited barrier facility for specific-pathogen-free mice in Tsinghua University. Mice were euthanized immediately upon being taken out of isolators. Animals were used according to protocols approved by the Institutional Animal Care and Use Committee.

### Plasmids

Human Rip2 cDNA was cloned from a cDNA library and inserted into pEGFP-N1 vector. Two mutants, S176A and K47M, were generated through site-directed mutagenesis. Myc-tagged human LRRK2 was as described (3). Myc-tagged human LRRK2 (K1906M) was from Dr. DC Rubinsztein. pSpCas9-2A-Puro-MCS and EZ-Guide XH vector were from Dr. Wei Gu. All the constructs were confirmed by sequencing.

### Antibodies

The following primary antibodies were used: anti-p38, anti-phosphorylated p38, anti-ERK1/2, anti-phosphorylated ERK1/2, anti-IκBα, anti-phosphorylated IκBα, anti-P65, anti-phosphorylated P65, anti-Tak1, and anti-myc antibody were from Cell Signaling Technology. Anti-phosphorylated Rip2 was from Abgent. Anti-JNK antibody, anti-phosphorylated JNK antibody, and anti-phosphorylated Tak1 were from Abcam. Anti-Rip2 antibody was from Abnova (H0000876-M02). Anti-LRRK2 antibody was from Epitomics (MJFF2 c41-2). Anti-actin antibody was from Sigma (A5441). Anti-GFP antibody was from Thermo Fisher Scientific (A-11120).

### Reagents

All the chemical reagents were from Sigma, unless otherwise specified. MDP, iE-DAP, and LPS were purchased from Invivogen. FBS, RPMI1640 and DMEM were from Life Technology.

### Cell culture and transfection

HEK293T cells were cultured in DMEM supplemented with 10% FBS and 1% penicillin/streptomycin at 37°C in 5% CO_2_ in a humidified incubator. THP-1 cells were cultured in RPMI 1640 supplemented with 10% FBS and 1% penicillin/streptomycin.

BMDM cells were cultured as described (Liu et al., 2011). Briefly, bone marrow cells from the femurs of C57/B6 mice were seeded into plates in DMEM supplemented with 10% FBS, 1% penicillin/streptomycin, 20 ng/mL M-CSF (Peptotech). After 3 days, fresh medium was added to the plate. The cells were ready for use on day 6.

### LRRK2 knockout THP-1 cell lines

The CRISPR/Cas9 system was used to knock out LRRK2 in THP-1 cells. LRRK2 guide RNA sequences were designed using online target prediction (http://crispr.mit.edu/). Two gRNA sequences, gRNA1 and gRNA2, were chosen to knock out LRRK2 in THP-1 cells. DNA oligos encoding the corresponding gRNA sequences were inserted into the cloning sites of pSpCas9-2A-Puro-MCS and the construct was transfected into THP-1 cells using a nucleofector (Amaxa). The transfected cells were subsequently treated with 0.8 μg/mL puromycin for 15 days. The effect of knockout was determined by immunoblotting. The sequences of gRNAs are as follows: gRNA 1(F), 5′-CACCGAGAAACGCTGGTCCAAATCCTGG-3′; gRNA 1(R), 5′-AAACCCAGGATTTGGACCAGCGTTTCTC-3′; gRNA 2(F), 5′-CACCGTGAACACCAGCAGATCCTCCAGG-3′; gRNA 2(R), 5′-AAACCCTGGAGGATCTGCTGGTGTTCAC-3′.

### Stimulation of BMDMs or THP-1 cells

Cultured BMDMs or THP-1 cells were treated with 5 ng/mL LPS and MDP (1 μg/ mL and 10 μg/mL) for 4 h before cells were harvested for total RNA extraction. BMDMs or THP-1 cells were treated with 5 ng/mL LPS and MDP (1 μg/mL and 10 μg/mL) for 12 h before supernatants were harvested. For ER stress-induced inflammation, cultured BMDMs were stimulated with thapsigargin (1 μmol/L and 10 μmol/L) for 24 h before cells were harvested for RNA extraction and supernatants were collected for ELISA assay.

### Isolation and quantification of mRNA

RNA from treated BMDMs or THP-1 cells was extracted with Trizol following the manufacturer’s instructions (Invitrogen). cDNA was transcribed using a PrimeScript RT reagent kit (Takara). Quantitative PCR (Q-PCR) reactions were performed using Light Cycler SYBR green DNA master mix (Takara) on an ABI7500 thermal cycler in triplicate. The specificity of Q-PCR was verified with melting curves of each PCR reaction. The level of target mRNA was determined by the difference of cycle threshold (Ct) values between the target and loading control. The level of GAPDH mRNA was used as the control to normalize loading. Q-PCR primers are listed below:

**Table:** 

Primer name	Primer sequence
mouse IL-6-F	ctctgggaaatcgtggaaat
mouse IL-6-R	ccagtttggtagcatccatc
mouse IL-1β-F	gtgctcatgtcctcatcctg
mouse IL-1β-R	cacagcagcacatcaacaag
mouse TNF-α-F	atgagaagttcccaaatggc
mouse TNF-α-R	ctccacttggtggtttgcta
mouse GAPDH-F	aaggtcatcccagagagctgaa
mouse GAPDH-R	ctgcttcaccaccttcttga
human IL-6-F	gtagccgccccacacaga
human IL-6-R	catgtctcctttctcagggctg
human IL-1β-F	aaatacctgtggccttgggc
human IL-1β-R	tttgggatctacactctccagct
human TNF-α-F	cccagggacctctctctaatca
human TNF-α-R	gcttgagggtttgctacaacatg
human GAPDH-F	aatcccatcaccatcttcca
human GAPDH-R	tggactccacgacgtactca

### Measurement of cytokines in supernatants

Supernatants from BMDMs were harvested. The concentrations of IL-6, IL-1β, and TNF-α in the supernatants were determined by ELISA kits (Biolegend) according to the manufacturer’s instructions.

### Cell transfection and Western blotting analysis

HEK293T cells were seeded at a concentration of 1 × 10^5^ cells/well and cultured overnight in 12-well plates. Transfections were performed with lipofectamine 2000 (Invitrogen) according to the manufacturer’s instructions together with various DNA constructs. Transfected cells were harvested and lysed with pre-chilled RIPA buffer containing protease inhibitor cocktail (Roche) and phosphatase inhibitor cocktail (Roche) on ice for 30 min. Supernatants were obtained by centrifugation at 10,000 g for 10 min at 4 ºC and subjected to immunoblotting.

### Immunoprecipitation

5 × 10^6^ HEK293T cells or THP-1 cells were harvested and lysed in 400 μL IP buffer (50 mM Tris-HCl, pH7.4, 150 mmol/L NaCl, 1% NP-40, 2 mmol/L EDTA) with protease inhibitor cocktail (Roche), 1 mmol/L PMSF, 10 mmol/L NaF, 1 mmol/L Na_3_VO_4_ on ice for 30 min. Supernatants were collected by centrifugation at 12,000 rpm for 10 min at 4°C. The supernatants were precleared with 20 μL DYNAbeads (Invitrogen) by rotating for 20 min at 4°C. The precleared supernatants were incubated with immunoprecipitation antibody by rotating for 3 h at 4°C. 20 μL DYNAbeads were added to the mixture of antibody and supernatant at 2 h. DYNAbeads was then washed three times with 500 μL immunoprecipitation buffer before being boiled in 25 μL SDS sample loading buffer (2×). Both input and immunoprecipitated proteins were subjected for immunoblotting analysis.

### Luciferase reporter assay

HEK293T cells were seeded at a concentration of 5 × 10^4^ cells/well and cultured overnight in 24-well plates. Cells were transfected with luciferase reporter plasmids using lipofectamine 2000. Thirty-six hours after transfection, luciferase activities were measured using the dual-luciferase kit (Promega).

## Electronic supplementary material

Below is the link to the electronic supplementary material.

Supplementary material 1 (PDF 332 kb)

## References

[CR1] Bertrand MJ, Doiron K, Labbe K, Korneluk RG, Barker PA, Saleh M (2009). Cellular inhibitors of apoptosis cIAP1 and cIAP2 are required for innate immunity signaling by the pattern recognition receptors NOD1 and NOD2. Immunity.

[CR2] Caruso R, Warner N, Inohara N, Nunez G (2014). NOD1 and NOD2: signaling, host defense, and inflammatory disease. Immunity.

[CR3] Damgaard RB, Nachbur U, Yabal M (2012). The ubiquitin ligase XIAP recruits LUBAC for NOD2 signaling in inflammation and innate immunity. Mol Cell.

[CR4] Dorsch M, Wang A, Cheng H (2006). Identification of a regulatory autophosphorylation site in the serine-threonine kinase RIP2. Cell Signal.

[CR5] Franke A, McGovern DP, Barrett JC (2010). Genome-wide meta-analysis increases to 71 the number of confirmed Crohn’s disease susceptibility loci. Nat Genet.

[CR6] Gardet A, Benita Y, Li C (2010). LRRK2 is involved in the IFN-gamma response and host response to pathogens. J Immunol.

[CR7] Gillardon F (2009). Leucine-rich repeat kinase 2 phosphorylates brain tubulin-beta isoforms and modulates microtubule stability–a point of convergence in Parkinsonian neurodegeneration?. J Neurochem.

[CR8] Hitotsumatsu O, Ahmad RC, Tavares R (2008). The ubiquitin-editing enzyme A20 restricts nucleotide-binding oligomerization domain containing 2-triggered signals. Immunity.

[CR9] Hsu CH, Chan D, Greggio E (2010). MKK6 binds and regulates expression of Parkinson’s disease-related protein LRRK2. J Neurochem.

[CR10] Imai Y, Gehrke S, Wang HQ (2008). Phosphorylation of 4E-BP by LRRK2 affects the maintenance of dopaminergic neurons in *Drosophila*. Embo J.

[CR11] Ito G, Katsemonova K, Tonelli F (2016). Phos-tag analysis of Rab10 phosphorylation by LRRK2: a powerful assay for assessing kinase function and inhibitors. Biochem J.

[CR12] Jaleel M, Nichols RJ, Deak M (2007). LRRK2 phosphorylates moesin at threonine-558: characterization of how Parkinson’s disease mutants affect kinase activity. Biochem J.

[CR13] Jostins L, Ripke S, Weersma RK (2012). Host-microbe interactions have shaped the genetic architecture of inflammatory bowel disease. Nature.

[CR14] Keestra-Gounder AM, Byndloss MX, Seyffert N (2016). NOD1 and NOD2 signalling links ER stress with inflammation. Nature.

[CR15] Kobayashi K, Inohara N, Hernandez LD (2002). RICK/Rip2/CARDIAK mediates signalling for receptors of the innate and adaptive immune systems. Nature.

[CR16] Kubo M, Nagashima R, Ohta E (2016). Leucine-rich repeat kinase 2 is a regulator of B cell function, affecting homeostasis, BCR signaling, IgA production, and TI antigen responses. J Neuroimmunol.

[CR17] Leszek J, Barreto GE, Gasiorowski K, Koutsouraki E, Avila-Rodrigues M, Aliev G (2016). Inflammatory mechanisms and oxidative stress as key factors responsible for progression of neurodegeneration: role of brain innate immune system. CNS Neurol Disord Drug Targets.

[CR18] Liu Z, Lee J, Krummey S, Lu W, Cai H, Lenardo MJ (2011). The kinase LRRK2 is a regulator of the transcription factor NFAT that modulates the severity of inflammatory bowel disease. Nat Immunol.

[CR19] Lytton J, Westlin M, Hanley MR (1991). Thapsigargin inhibits the sarcoplasmic or endoplasmic reticulum Ca-ATPase family of calcium pumps. J Biol Chem.

[CR20] Magalhaes JG, Lee J, Geddes K, Rubino S, Philpott DJ, Girardin SE (2011). Essential role of Rip2 in the modulation of innate and adaptive immunity triggered by Nod1 and Nod2 ligands. Eur J Immunol.

[CR21] McGeer PL, Itagaki S, Boyes BE, McGeer EG (1988). Reactive microglia are positive for HLA-DR in the substantia nigra of Parkinson’s and Alzheimer’s disease brains. Neurology.

[CR22] Nakamura N, Lill JR, Phung Q (2014). Endosomes are specialized platforms for bacterial sensing and NOD2 signalling. Nature.

[CR23] Paisan-Ruiz C, Jain S, Evans EW (2004). Cloning of the gene containing mutations that cause PARK8-linked Parkinson’s disease. Neuron.

[CR24] Park JH, Kim YG, McDonald C (2007). RICK/RIP2 mediates innate immune responses induced through Nod1 and Nod2 but not TLRs. J Immunol.

[CR25] Peterson LW, Artis D (2014). Intestinal epithelial cells: regulators of barrier function and immune homeostasis. Nat Rev Immunol.

[CR26] Philpott DJ, Sorbara MT, Robertson SJ, Croitoru K, Girardin SE (2014). NOD proteins: regulators of inflammation in health and disease. Nat Rev Immunol.

[CR27] Rioux JD, Xavier RJ, Taylor KD (2007). Genome-wide association study identifies new susceptibility loci for Crohn disease and implicates autophagy in disease pathogenesis. Nat Genet.

[CR28] Rocha NP, de Miranda AS, Teixeira AL (2015). Insights into neuroinflammation in Parkinson’s disease: from biomarkers to anti-inflammatory based therapies. Biomed Res Int.

[CR29] Stafa K, Trancikova A, Webber PJ, Glauser L, West AB, Moore DJ (2012). GTPase activity and neuronal toxicity of Parkinson’s disease-associated LRRK2 is regulated by ArfGAP1. PLoS Genet.

[CR30] Suzuki K, Akama T, Kawashima A, Yoshihara A, Yotsu RR, Ishii N (2012). Current status of leprosy: epidemiology, basic science and clinical perspectives. J Dermatol.

[CR31] Wandu WS, Tan C, Ogbeifun O (2015). Leucine-rich repeat kinase 2 (Lrrk2) deficiency diminishes the development of experimental autoimmune uveitis (EAU) and the adaptive immune response. PLoS One.

[CR32] Yang Y, Yin C, Pandey A, Abbott D, Sassetti C, Kelliher MA (2007). NOD2 pathway activation by MDP or Mycobacterium tuberculosis infection involves the stable polyubiquitination of Rip2. J Biol Chem.

[CR33] Zhang FR, Huang W, Chen SM (2009). Genomewide association study of leprosy. N Engl J Med.

[CR34] Zhang Q, Pan Y, Yan R (2015). Commensal bacteria direct selective cargo sorting to promote symbiosis. Nat Immunol.

[CR35] Zhu Y, Wang C, Yu M, Cui J, Liu L, Xu Z (2013). ULK1 and JNK are involved in mitophagy incurred by LRRK2 G2019S expression. Protein Cell.

[CR36] Zimprich A, Biskup S, Leitner P (2004). Mutations in LRRK2 cause autosomal-dominant Parkinsonism with pleomorphic pathology. Neuron.

