# Supplementary material for: LRRK2 enhances Nod1/2-mediated inflammatory cytokine production by promoting Rip2 phosphorylation
Source: Protein Cell. 2016 Nov 9;8(1):55–66. doi: 10.1007/s13238-016-0326-x (PMC5233611; doi:10.1007/s13238-016-0326-x)
Supplement: Supplementary file 1 — Supplementary material 1 (PDF 332 kb) [file 13238_2016_326_MOESM1_ESM.pdf]

Supplementary Figures

Supplementary Figure 1

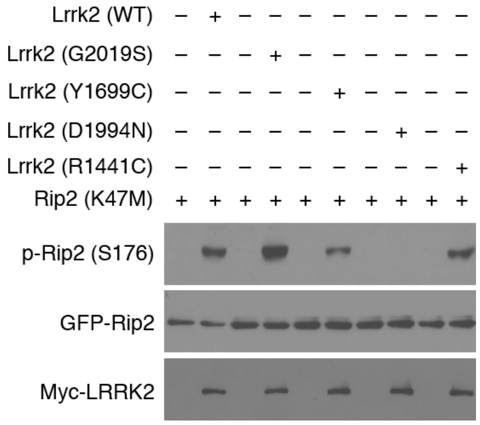

Supplementary Figure 1 Differential effects of LRRK2 mutants (G2019S, Y1699C, and R1441C) on the phosphorylation of Rip2. The level of phosphorylated Rip2 at residue of Ser 176 was immunoblotted in HEK293T transfected with LRRK2 variants (WT, G2019S, Y1699C, D1994N, and R1441C) together with Rip2 kinase death mutant (K47M). The levels of total Rip2 and LRRK2 were analyzed using immunoblotting. The data are representative from three independent experiments.

Supplementary Figure 2

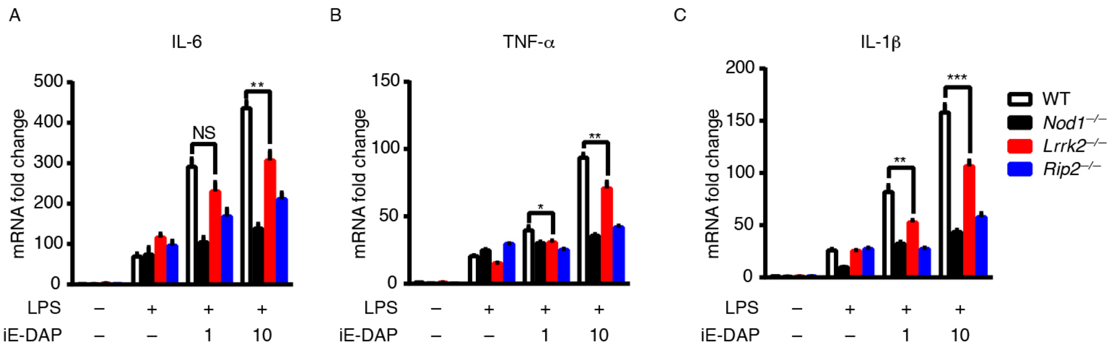

Supplementary Figure 1 LRRK2 enhances cytokine production in iE-DAP-treated macrophages. (A-C) The relative fold changes of IL-6 (A), TNF-α (B), IL-1β (C) mRNA transcripts in WT, *Nod1*$^{-/-}$, *Rip2*$^{-/-}$ and *Lrrk2*$^{-/-}$ BMDMs treated with 5 ng/ml LPS and 1 or 10 μg/ml iE-DAP for 4 hours, or mock-treated. Data are expressed as mean ± s.e.m. $*P < 0.05$, $**P < 0.01$, $***P < 0.001$, NS denotes not significant, Student's t-test. The data are representative from three independent experiments.
